# Assessment of HER2 Status Using Immunohistochemistry (IHC) and Fluorescence *In Situ* Hybridization (FISH) Techniques in Mucinous Epithelial Ovarian Cancer: A Comprehensive Comparison between ToGA Biopsy Method and ToGA Surgical Specimen Method

**DOI:** 10.1371/journal.pone.0142135

**Published:** 2015-11-13

**Authors:** Wan-Ru Chao, Ming-Yung Lee, Alexandra Ruan, Huang Pin Sheng, Jeng-Dong Hsu, Chih-Ping Han, Chiew-Loon Koo

**Affiliations:** 1 Department of Pathology, Chung-Shan Medical University and Chung Shan Medical University Hospital, Taichung, Taiwan; 2 Department of Statistics and Informatics Science, Providence University, Taichung, Taiwan; 3 Keck School of Medicine of the University of Southern California, Los Angeles, California, United States of America; 4 Department of Obstetrics and Gynecology, School of Medicine, Chung-Shan Medical University and Chung-Shan Medical University Hospital, Taichung, Taiwan; 5 Department of Pathology, Tungs’ Taichung MetroHarbor Hospital, Taichung, Taiwan; INRS, CANADA

## Abstract

We aimed to compare the assay performance characteristics of HER2 status in mucinous epithelial ovarian cancer (EOC) by ToGA (Trastuzumab for Gastric Cancer) biopsy versus ToGA surgical specimen methods. Forty-nine tissue microarray (TMA) samples of mucinous EOC from Asian women were analyzed by immunohistochemistry (IHC) and fluorescence in situ hybridization (FISH) tests using ToGA trial HER2 scoring methods. The overall concordance between IHC and FISH by the ToGA surgical specimen method is 97.56% and by the ToGA biopsy specimen method is 97.14%. The agreements of HER2 IHC results under both biopsy and surgical specimen methods were nearly perfect (weighted kappa = 0.845). Additionally, the percentage of *Her2* FISH amplification showed increasing trend with increasing HER2 IHC ordinals (negative, equivocal, positive) by both TOGA biopsy (P<0.001) and surgical specimen method (P<0.001). After excluding equivocal cases, the sensitivity (100%), PPV (88.89%) and NPV (100%) of HER2 IHC were unchanged under either surgical specimen method or biopsy method. However, the specificity (96.97%) and accuracy (97.56%) of HER2 IHC was slightly higher under the surgical specimen method than those (specificity 96.30%, accuracy 97.14%) under the biopsy method. Of the total 49 cases, the number (n = 14) of HER2 IHC equivocal results under the ToGA biopsy method was 1.75-fold higher than those (n = 8) under the ToGA surgical specimen method (28.57% vs. 16.32%). Therefore, compared to ToGA surgery specimen method, the ToGA biopsy method caused more equivocal IHC cases to be referred to FISH testing and did not increase the detection rates of *Her2* FISH amplification.

## Introduction

The encouraging success of trastuzumab and new anti-HER2 therapies in breast, gastric or gastro-oesophageal junction (GEJ) cancer patients has prompted us to investigate the HER2 status and its possible therapeutic implication in other malignancies, including mucinous EOC [1,2]. Even though the methods currently used to assess HER2 status are well-established in breast cancer and gastric/GEJ cancers, there has been no standardization of HER2 scoring in mucinous EOC so far.

Both gastric cancer and mucinous EOC share similar cell morphology, apical mucin secretion, glandular architecture and characteristic HER2 immunostaining patterns, while breast cancer typically does not share these characteristics. It would be more realistic to adopt the Trastuzumab for Gastric Cancer (ToGA) trial scoring method rather than The American Society of Clinical Oncology and the College of American Pathologists (ASCO/CAP) guideline recommendations as a reference guide in assessing the HER2 status in our cohort of mucinous ovarian cancer.

Using the TMA and ToGA surgical specimen scoring method, we previously reported the positive correlation between *Her2* gene copy numbers and HER2 protein expressions in mucinous EOC [3]. However, the size of core tissues in the TMA were more comparable to that of the biopsy specimen, so using the ToGA biopsy scoring method seems to be more representative.

In this study, we planned to elucidate and compare the assay performance characteristics of the two ToGA (biopsy vs. surgical specimen) methods for HER2 reporting in mucinous EOC.

## Materials and Methods

All patient-derived study materials were collected and archived under the protocol approved by the institutional review board at Chung-Shan Medical University Hospital (IRB No: CS11008 & CS13239) that met the current guidelines of the Taiwan Government’s Ministry of Health and Welfare (MOHW) and Forum for Ethical Review Committees in Asia and the Western Pacific (FERCAP). All the organizations and operations of this institutional review board are in compliance with ICH-GCP (International Conference on Harmonization—Good Clinical Practice) requirements and the essence of the Declaration of Helsinki. Their characteristics of the TMA derivation were described in our previous report [3]. Briefly, we had 2 sources of the 49 cases of de-linked mucinous EOC.

1. The first group of 21 cases of Taiwanese mucinous EOC consisted of selected formalin-fixed, paraffin-embedded oophorectomy tissue blocks retrieved from the archives of the Tissue Bank of Clinical Trial Center, Chung-Shan Medical University Hospital.
2. The second group of 28 cases of Asian mucinous EOC was derived from a commercial set of TMA slides (US Biomax Inc Catalog No. Ov2001). Of all 200 cores in this TMA, there were only 28 cores of mucinous EOC available for this study.

All direct patient identifiers had already been permanently deleted so that we were not able to obtain their agreement for use in this study. The reason for lack of consent was that the identity and personal information of an individual who has donated human tissue is no longer identifiable or linked to that individual’s tissue sample.

### Immunohistochemistry (IHC)

The HER2 immunostains were performed on the fully automated Ventana Benchmark XT autostainer using pathway antiHER2/neu rabbit monoclonal antibody (4B5, Ventana medical system Inc). HER2 IHC score 3+ breast cancer was used as a positive control. Negative controls were obtained by excluding the primary antibody. The slides were mounted with Permount for microscopic examination, and the images were captured by the NIKON ECLIPSE 50i microscope and NIKON DS-Fi1 Digital Camera System for study comparison.

### Fluorescence in situ hybridization (FISH)

The FISH test was performed by the ABBOTT/Vysis PathVysion *Her2* DNA Probe Kit protocol (Path-Vysion CE Product Description, 4/29/2008). The dual-color FISH consists of two labeled DNA probes and was performed on sections cut from the same tissue microarrays (TMA) block. The LSI HER2 probe that spans the entire *Her2* gene was labeled in Spectrum Orange and the CEP17 (chromosome-17 centromere; for chromosome-17 enumeration) probe was labeled in SpectrumGreen then hybridized to the alpha satellite DNA located at the centromere of chromosome-17 (17p11.1–q11.1). Counting two separate fields of at least 20 cells was essential. We calculated the *Her2*:CEP17 signal ratio by recording the numbers of *Her2* gene (red) and chromosome 17 (green) signals from preselected tumor areas. In most cases, tumor cells from matching sites of IHC analysis were scored for the number of red (*Her2*) and green (CEP17) signals. Signal photos were taken with the NIKON ECLIPSE 80i fluorescent microscope with a PlanFluor oil objective (100x) using a double band-pass filter that permits simultaneous green and red color penetration.

### IHC and FISH interpretation

**Fig 1:**
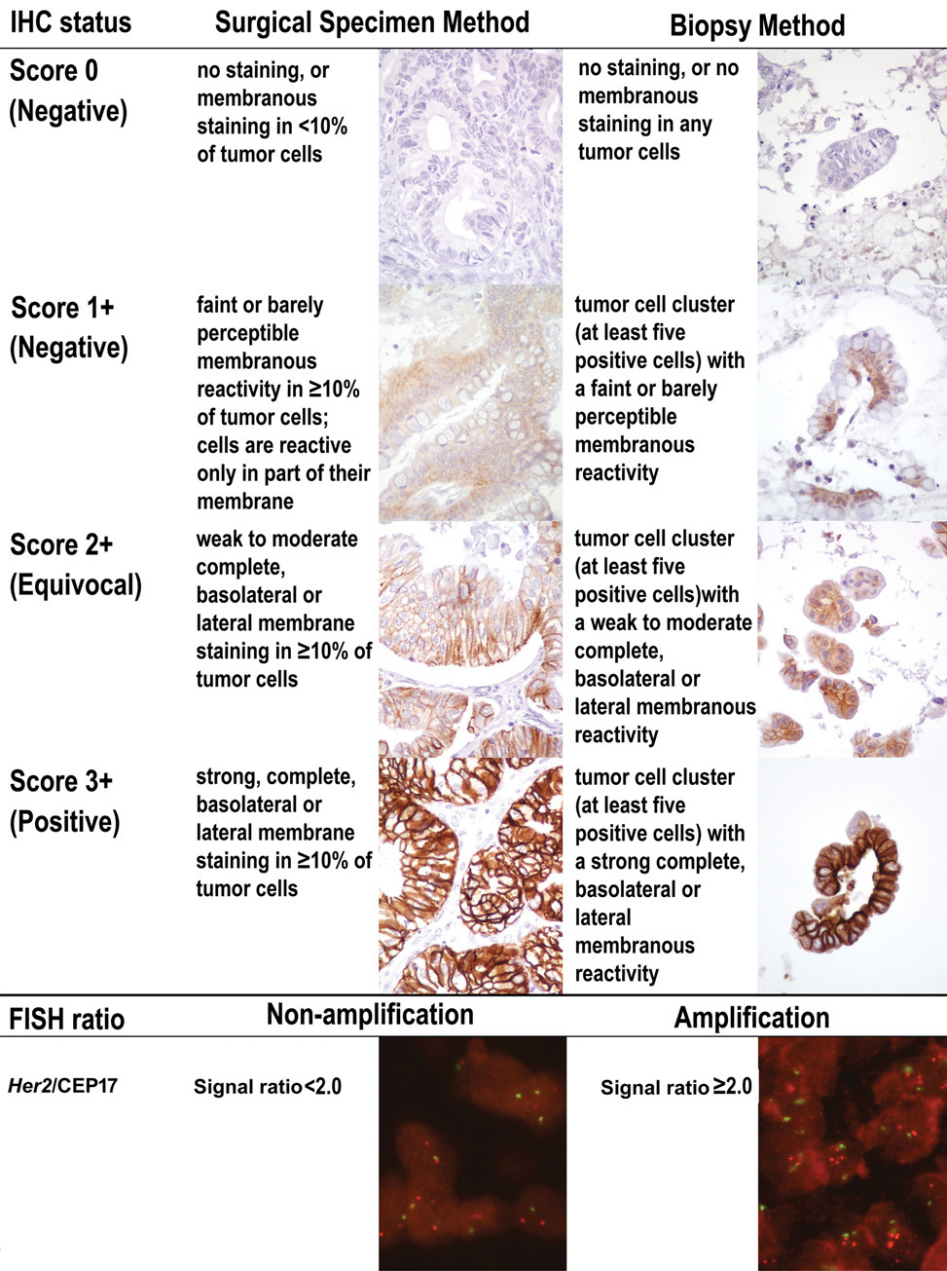
HER2 IHC and *Her2* FISH testing results by the ToGA surgical specimen and ToGA biopsy scoring methods in mucinous EOC.

We applied both the ToGA surgical specimen and ToGA biopsy scoring methods to interpret the HER2 IHC and *Her2* FISH testing results in this study. (Fig 1)

For quality assurance and quality control (QA/QC) assessments, our laboratory performance met the proficiency testing (PT) requirements of the Taiwan Division of International Academy of Pathology. Additionally, we participated in the UK NEQS (United Kingdom National External Quality Assessment Scheme) HER2 test program. We also ran a HER2 control in all cases daily and had one pathologist routinely screen the slides.

### Statistical analysis

The consistency between ToGA surgical specimen and ToGA biopsy IHC results were analyzed by categorized variables by weighted Kappa statistics. The strength of agreement beyond chance for different ranges were referred from the report of Landis and Koch [4]. Furthermore, we applied the Cochran-Armitage trend test to assess a trend of positive percentage across the ordinal IHC categories. Utilizing FISH as the reference standard, sensitivity was defined as the ratio of HER2 IHC positive cases among *Her2* FISH amplified patients, specificity was defined as the ratio of HER2 IHC negative cases among *Her2* FISH non-amplified patients, positive predictive value (PPV) was defined as the ratio of *Her2* FISH amplified cases among HER2 IHC positive patients, negative predictive value (NPV) was defined as the ratio of *Her2* FISH non-amplified cases among HER2 IHC negative patients, and accuracy was defined as the ratio of HER2 IHC positive and *Her2* FISH amplified cases plus HER2 IHC negative cases and *Her2* FISH non-amplified cases among all cases. The overall concordance was defined as the ratio of HER2 IHC positive and *Her2* FISH amplified cases plus HER2 IHC negative cases and *Her2* FISH non-amplified cases among all non-equivocal IHC cases. Data were analyzed using standard statistical software (SPSS, Inc., Chicago, IL). All tests were 2-sided and the significance level was 0.05.

## Results

In this study, a total of 49 specimens of mucinous EOC from Asian women were available for the evaluation of HER2 immunostaining status by both the ToGA surgical specimen and ToGA biopsy methods. Six IHC negative cases under the ToGA surgical specimen method were reclassified as IHC equivocal cases by the ToGA biopsy method. Twenty-six IHC negative cases, eight IHC equivocal cases and nine IHC positive cases were unchanged by either ToGA surgical specimen method or ToGA biopsy method. Both the 3-tier HER2 IHC categories under respective methods exhibited almost perfect agreement (surgical specimen *vs*. biopsy; linear weighted kappa = 0.845, 95% CI 0.7226–0.9676). (Table 1)

**Table 1:** Concordance of HER2 IHC results derived from ToGA surgical specimen versus ToGA biopsy scoring criteria by Kappa statistics.

HER2 IHC ToGA biopsy	ToGA surgical specimen			
	Negative	Equivocal	Positive	Total
Negative	26	0	0	26
Equivocal	6	8	0	14
Positive	0	0	9	9
total	32	8	9	49

Linear Weighted Κappa = 0.845, 95%CI 0.7226–0.9676

We demonstrated that the *Her2* amplification rate of mucinous EOC was 20.41% (n = 10/49). Additionally the percentage of *Her2* FISH amplifications increased significantly in a trend through the order of HER2 IHC results (negative, equivocal, positive) by both ToGA surgical specimen method and ToGA biopsy method, respectively (P<0.001; P<0.001) (Table 2). Under the ToGA surgical specimen method, eight cases were classified as HER2 IHC score 2+ (equivocal), which consisted of six cases (75.00%) of *Her2* gene non-amplification (n = 6/8) and two cases (25.00%) of *Her2* gene amplification (n = 2/8). Under the ToGA biopsy method, fourteen cases were classified as IHC score 2+ (equivocal), which consisted of twelve cases (85.71%) of *Her2* gene non-amplification (n = 12/14) and the same two cases (14.29%) of *Her2* gene amplification (n = 2/14). (Table 2)

**Table 2:** Her2 FISH ratio results and HER2 immunohistochemical results by both the ToGA surgical specimen and the ToGA biopsy scoring criteria, respectively.

	ToGA surgical specimen				
	HER2 IHC				
*Her2* FISH*	Negative	Equivocal	Positive	total	P
Non-amplified	32(100%)	6(75%)	1(11.11%)	39(79.59%)	<0.001^¥^
amplified	0	2(25%)	8(88.89%)	10(20.41%)	
total	32	8	9	49	
	ToGA biopsy specimen				
	HER2 IHC				
*Her2* FISH*	Negative	Equivocal	Positive	total	P
Non-amplified	26(100%)	12(85.71%)	1(11.11%)	39(79.59%)	<0.001^¥^
amplified	0	2(14.29%)	8(88.89%)	10(20.41%)	
total	26	14	9	49	

*no equivocal FISH result in ToGA

^¥^Cochran-Armitage trend test

Except for 8 HER2 IHC equivocal cases by the ToGA surgical specimen method, our data for the relationship between IHC and FISH showed 88.89% (n = 8/9) in positive concordance, 100% (n = 32/32) in negative concordance and 97.56 (n = 40/41) in overall concordance (kappa = 0.9259); while except for 14 HER2 IHC equivocal cases by the ToGA biopsy method, our data for the relationship between IHC and FISH showed 88.89% (n = 8/9) in positive concordance, 100% (26/26) in negative concordance and 97.14% (34/35) in overall concordance (kappa = 0.9224). Additionally, we identified the one case who showed positive HER2 IHC/ non-amplified *Her2* FISH by both methods. (Table 3)

**Table 3:** HER2 status in mucinous EOCs: Concordance rates between IHC and FISH by both the ToGA surgical specimen and biopsy criteria.

ToGA surgical specimen criteria				
	HER2 IHC^¥^			
*Her2* FISH*	Negative	Positive	total	Kappa
Non-amplified	32(100%)	1(11.11%)	33	0.9259
amplified	0	8(88.89%)	8	(0.782,1)
total	32	9	41	
ToGA biopsy specimen criteria				
	HER2 IHC^¥^			
*Her2* FISH*	Negative	Positive	total	Kappa
Non-amplified	26(100%)	1(11.11%)	27	0.9224
amplified	0	8(88.89%)	8	(0.773,1)
total	26	9	35	

*No equivocal FISH category existed by the ToGA criteria

¥Equivocal (IHC 2+) cases by the ToGA criteria were excluded from Concordance and Kappa statistics

Negative indicated HER2 IHC score 0, 1+.

Positive indicated HER2 IHC score 3+

Using *Her2* FISH as the reference standard, the HER2 IHC performance characteristics under the ToGA surgical specimen and biopsy methods were evaluated by calculation of sensitivity, specificity, positive predictive values (PPV), negative predictive value (NPV) and accuracy. After excluding equivocal cases, the sensitivity (100%), PPV (88.89%) and NPV (100%) by both ToGA surgical specimen and ToGA biopsy methods are similar. The accuracy under the ToGA surgical specimen method was slightly higher than that under the ToGA biopsy method (97.56% *vs*. 97.14%) and the specificity was also (96.97% *vs*. 96.30%) slightly increased (Table 4).

**Table 4:** HER2 IHC performance measures by both ToGA surgical specimen and ToGA biopsy scoring criteria respectively.

	Sensitivity	Specificity	PPV	NPV	accuracy
	(95%CI)	(95%CI)	(95%CI)	(95%CI)	(95%CI)
ToGA surgical specimen(n = 41)	100%	96.97%	88.89%	100%	97.56%
	(100%, 100%)	(96.46%, 97.48%)	(85.40%, 92.38%)	(100%, 100%)	(97.19%, 97.93%)
ToGA biopsy (n = 35)	100%	96.30%	88.89%	100%	97.14%
	(100%, 100%)	(95.61%, 96.99%)	(85.40%, 92.38%)	(100%, 100%)	(96.67%, 97.61%)

## Discussion

Currently, IHC and FISH are still the most widely used methods for assessing HER2 status in clinical specimens, but there have been debates about their relative merits for use in various cancers [5]. Yoon et al. proposed an algorithm for esophageal cancer that puts IHC up front, with FISH restricted to cases with an equivocal IHC [6]. Rüschoff stated again that IHC testing should be the primary method of choice to determine HER2 status in gastric cancer, and limit the use of FISH to those cases that have equivocal HER2 expression [7]. Of both the IHC and FISH assay methods, the 2013 ASCO/CAP guideline advised that either one could be used first in breast cancer, and followed later on by another one, whenever the forerunning testing showed equivocal results [8]. Applying both the 2007 and 2013 ASCO/CAP breast cancer guidelines, we have recently reported their appraisals of HER2 status in mucinous EOC [9,10]. However, neither a standard nor consensus for the interpretation of HER2 IHC and *Her2* FISH results in mucinous EOCs exist as of yet.

In this study, we adopted the ToGA trial scoring methods to investigate the HER2 status in mucinous EOC. The reasons were that both gastric adenocarcinoma and ovarian mucinous EOC had similar morphologies of incomplete, lateral/basolateral U shape HER2 immunostaining with luminal face sparing, which is rarely seen in breast cancer [3].

There is no equivocal *Her2* FISH result category by the classification under the ToGA criteria [2]. Instead, only two subgroups (amplification vs. non-amplification) were divided under *Her2* FISH analysis. Moreover, the DNA target is more stable and easier to interpret. Calculating the *Her2* gene number has less inter-observer variation. These findings supported the use of use *Her2* FISH as a reference standard in this study. Accordingly, through overall consideration of the practice convenience and cost effectiveness, it was reasonable to propose a method to evaluate the HER2 status in mucinous EOC where IHC is used for initial screening, and FISH testing is restricted to cases with equivocal IHC results.

Previously, we reported that both CEP17 corrected and uncorrected *HER2* gene copies correlated significantly with HER2 IHC results by the ToGA “surgical specimen” method of evaluating mucinous EOC [3]. However, we used the Ventana Medical Systems (Tucson, AZ) 4B5 antibody in this study instead of the DAKO A0485 antibody (Carpinteria, CA), since the 4B5 assay showed evidence of superior performance in comparison to the A0485 assay [11]. Additionally, whether the core tissues in TMAs are comparable to biopsy samples has also been a debatable issue. As a result, we compared the assay performance of both ToGA methods (biopsy *vs*. surgical specimen) in this study.

The major differences of HER2 IHC between ToGA surgical (large sample) and ToGA biopsy (small sample) scoring methods are noted as below:

1. A tumor is classified as HER2 protein over-expression by IHC positive (score 3+) when there is strong, complete, basolateral or lateral membrane staining in ≥10% of tumor cells in surgical specimen *versus* a cluster of at least five positive cells in a biopsy specimen. (Fig 1) Our data exhibited 9 cases of HER2 IHC positive (score 3+) by both ToGA surgical and ToGA biopsy methods. (Tables 1 and 2)
2. An equivocal HER2 IHC test in ToGA surgical specimen was defined as weak to moderate complete, basolateral or lateral membrane staining in ≥10% of tumor cells *versus* a cluster of at least five positive tumor cells in a biopsy specimen. (Fig 1) Our data exhibited six cases of HER2 IHC negative (score 0, 1+) by the ToGA surgical specimen method that were upgraded to equivocal (score 2+) by the ToGA biopsy method. Eight cases remained the same ToGA classification as HER2 IHC equivocal (score 2+) by both ToGA surgical specimen and ToGA biopsy methods. (Table 1)

As demonstrated in this study, the percentage of *Her2* FISH amplifications increased significantly in a trend through their corresponding HER2 IHC ordinal categories by individual method, respectively (P<0.001, P<0.001). (Table 2) In addition, the overall non-equivocal concordance rates between HER2 IHC and *Her2* FISH were both almost perfect by both the ToGA surgical specimen method (n = 40/41; kappa = 0.9259) and biopsy method (n = 34/35; kappa = 0.9224). (Table 3) After reviewing IHC control slides and carefully excluding tissue processing and staining problems, we identified one case, showing positive HER2 IHC / non-amplified *Her2* FISH by both ToGA surgical specimen and TGA biopsy methods. The discordance rates between HER2 IHC and *Her2* FISH are comparable by the ToGA surgical (n = 1/41) and TGA biopsy (n = 1/35) methods (2.44% *vs*. 2.86%) (Table 3) This is the same one as has been described previously [3]. We suggest that discrepancies between HER2 protein overexpression and Her2 gene amplification might result from transcription, post-transcription modification or epigenetic dysregulation.

Of the total 49 cases, the number (n = 14) of HER2 IHC equivocal results under the ToGA biopsy method was 1.75-fold higher than those (n = 8) under the ToGA surgical specimen method (28.57% *vs*. 16.32%). (Table 1) Of those samples which were in the equivocal HER2 IHC category under both ToGA biopsy and ToGA surgical specimen methods, we recognized the same two cases who showed HER2 IHC equivocal (score 2+) and *Her2* FISH amplification by both methods. All the remaining 12 cases by the biopsy method and 6 cases by the surgical specimen method showed HER2 IHC equivocal (score 2+) and *Her2* FISH non-amplification. (Table 2) This indicates that the ToGA biopsy method caused more cases to be diagnosed as equivocal HER2 IHC, and therefore would need additional FISH testing to be performed.

Intratumoral heterogeneities of HER2 status have been previously reported in mucinous EOC [12,13]. Even though sampling with optimal cores in TMA can show accuracies when compared with whole mount sections, we predicted that the actual prevalence of intratumoral heterogeneity of HER2 status in our study would be underestimated due to the limited number of cases available for whole tissue block sections [3, 14, 15].

Using the *Her2* FISH as a reference standard, we evaluated the performance characteristics of HER2 IHC testing under both the ToGA surgical specimen and ToGA biopsy methods. (Table 4) Compared with the biopsy method, the ToGA surgical specimen method demonstrated superior specificity but comparable sensitivity, PPV and NPV. In particular, we identified that the ToGA surgical specimen method achieved better assay accuracy than biopsy method did in assessing HER2 status in mucinous EOC.

In summary, our data demonstrated that: (1) both ToGA biopsy (n = 34/35; 97.14%) and ToGA surgical specimen (n = 40/41; 97.56%) methods exhibit almost perfect concordance between nonequivocal IHC and FISH (kappa = 0.9224 for the biopsy method; kappa = 0.9259 for the surgical specimen method) in assessing HER2 status of mucinous EOC (Table 3), and (2) in comparison to the ToGA surgical specimen method, ToGA biopsy method caused more cases to be classified as Her2 IHC equivocal (n = 14 by the biopsy method *vs*. n = 8 by the surgical specimen method), but did not increase the detection rates of *Her2* FISH amplification (Table 2). As a result, we suggested that it would be reasonable to put the IHC by the ToGA surgical specimen method as the first step, with *Her2* FISH restricted to cases with an equivocal (score 2+) HER2 IHC on the basis of cost effectiveness in assessing the HER2 status in mucinous EOC.

## Conclusion

Identifying the exact HER2 status in mucinous EOC is essential for selecting potential candidates for novel anti-HER2 drug therapies. The ToGA surgical specimen method resulted in less equivocal demonstration of HER2 immunoreactivity; while the ToGA biopsy method caused more equivocal IHC cases to be referred to FISH testing than the ToGA surgery specimen method did. Additionally, the ToGA biopsy method using a cluster of greater than five positive cells rather than a percentage cut off did not increase the detection rate of *Her2* FISH amplification. Considering the higher equivocal rate and slightly lower accuracy under the ToGA biopsy method, we prefer to adopt the ToGA surgical specimen method rather than use ToGA biopsy method for the assessment of HER2 status in mucinous EOC.

## Supporting Information

S1 Table HER2 IHC scores and *Her2* FISH ratios for each case. We presented the dataset on HER2 IHC scores under both ToGA surgical specimen and ToGA biopsy methods and *Her2* FISH ratios of all 49 cases with primary mucinous EOC.
(DOCX)
