# Supplementary material for: Assessment of HER2 Status Using Immunohistochemistry (IHC) and Fluorescence In Situ Hybridization (FISH) Techniques in Mucinous Epithelial Ovarian Cancer: A Comprehensive Comparison between ToGA Biopsy Method and ToGA Surgical Specimen Method
Source: PLoS One. 2015 Nov 13;10(11):e0142135. doi: 10.1371/journal.pone.0142135 (PMC4643932; doi:10.1371/journal.pone.0142135)
Supplement: S1 Table — We presented the dataset on HER2 IHC scores under both ToGA surgical specimen and ToGA biopsy methods and Her2 FISH ratios of all 49 cases with primary mucinous EOC. (DOCX) [file pone.0142135.s001.docx]

**S1 Table.**

HER2 IHC scores and *Her2* FISH ratios for each case

| case | HER2 IHC score under  ToGA surgical specimen | HER2 IHC score under  ToGA biopsy | *Her2* FISH ratio |
| --- | --- | --- | --- |
| 1 | 0 | 0 | 0.93 |
| 2 | 3 | 3 | 1.58 |
| 3 | 1 | 1 | 1.50 |
| 4 | 3 | 3 | 6.90 |
| 5 | 0 | 1 | 1.69 |
| 6 | 1 | 1 | 1.77 |
| 7 | 0 | 0 | 1.35 |
| 8 | 0 | 0 | 1.46 |
| 9 | 3 | 3 | 9.90 |
| 10 | 2 | 2 | 1.57 |
| 11 | 0 | 0 | 1.18 |
| 12 | 0 | 1 | 1.25 |
| 13 | 0 | 1 | 1.38 |
| 14 | 0 | 1 | 1.33 |
| 15 | 0 | 1 | 1.14 |
| 16 | 0 | 0 | 1.50 |
| 17 | 1 | 2 | 1.38 |
| 18 | 1 | 2 | 1.20 |
| 19 | 0 | 0 | 1.07 |
| 20 | 0 | 1 | 1.12 |
| 21 | 1 | 2 | 1.00 |
| 22 | 1 | 1 | 0.94 |
| 23 | 0 | 0 | 1.19 |
| 24 | 0 | 1 | 1.06 |
| 25 | 0 | 0 | 1.27 |
| 26 | 1 | 2 | 1.06 |
| 27 | 1 | 1 | 1.00 |
| 28 | 0 | 0 | 1.00 |
| 29 | 3 | 3 | 4.30 |
| 30 | 0 | 0 | 0.92 |
| 31 | 0 | 0 | 0.89 |
| 32 | 2 | 2 | 2.90 |
| 33 | 1 | 1 | 1.03 |
| 34 | 1 | 2 | 1.03 |
| 35 | 1 | 1 | 1.02 |
| 36 | 2 | 2 | 0.95 |
| 37 | 0 | 1 | 1.15 |
| 38 | 0 | 1 | 0.90 |
| 39 | 2 | 2 | 1.30 |
| 40 | 3 | 3 | 6.30 |
| 41 | 2 | 2 | 0.93 |
| 42 | 2 | 2 | 1.00 |
| 43 | 1 | 2 | 1.00 |
| 44 | 2 | 2 | 1.14 |
| 45 | 2 | 2 | 1.14 |
| 46 | 3 | 3 | 6.00 |
| 47 | 3 | 3 | 5.54 |
| 48 | 3 | 3 | 5.83 |
| 49 | 3 | 3 | 3.33 |
